# Successful stabilisation of nephropathy in a patient with POEMS (polyneuropathy, organomegaly, endocrinopathy, M-band, skin changes) syndrome on treatment with mycophenolate and steroids: a case report

**DOI:** 10.1186/1752-1947-4-63

**Published:** 2010-02-22

**Authors:** Gareth J Rosser, Pablo Garcia Reitböck, Martin C Gray, Paul Warwicker

**Affiliations:** 1 Lister Renal Unit, Lister Hospital, Stevenage, SG1 4AB, UK; 2 Department of Cellular Pathology, Royal Free Hospital, London, NW3 2QG, UK

## Abstract

**Introduction:**

Renal involvement in POEMS (polyneuropathy, organomegaly, endocrinopathy, M-band, skin changes) syndrome is considered to be an under-diagnosed phenomenon with no clear treatment path. The limited literature suggests steroids to be the drug of choice, although improvements are limited and usually reverse on withdrawal of the drug.

**Case presentation:**

A 52-year-old Caucasian woman presenting with features consistent with POEMS syndrome developed progressive renal impairment with proteinuria. Renal biopsy revealed a membranoproliferative glomerulonephritis. She was treated with relatively low dose oral mycophenolate mofetil and prednisolone which stabilised her nephropathy and neuropathy.

**Conclusion:**

We describe an alternative therapeutic option in patients with this serious but poorly understood condition.

## Introduction

POEMS (polyneuropathy, organomegaly, endocrinopathy, M-band, skin changes) syndrome was first described by Nakanishi *et al. *[1] in Japan where it is known as Crow-Fakuse syndrome. A diagnosis of POEMS syndrome can be made if patients exhibit major criteria consisting of polyneuropathy and monoclonal plasmo-proliferative disorder in conjunction with at least one minor criterion from sclerotic bone lesions, Castleman disease, organomegaly, oedema, endocrinopathy, skin changes and papilloedema [2]. However, others have suggested that all these diagnostic criteria are not always evident [3,4].

Renal involvement in POEMS syndrome is recognised, but incidence is uncertain. An early report by Driedger and Pruzanski [5] suggested that 50% of patients demonstrated elevated blood urea nitrogen (BUN) or proteinuria, whereas Navis *et al. *[6] found that, of 200 patients with POEMS, only 21 had evidence of renal involvement.

Nakamoto *et al. *[7] reviewed 52 cases of Japanese patients with nephropathy. All patients manifested four or all of the five signs of POEMS. Polyneuropathy was present in all but one patient. A majority of cases had monoclonal protein in blood (either IgA- or IgG-) although 10 demonstrated a polyclonal hypergammaglobulinaemia, but in these, four other signs of POEMS supported the diagnosis. Additional signs included papilloedema, serositis with massive fluid retention, leg oedema, clubbed fingers, and cutaneous angiomas. Isolated plasmacytoma was present in four patients, and lesions in the remaining patients, if present, were of the osteosclerotic type. Castleman-like lymphoma was observed in nine patients. The median age of the patients was 49 years (range 28-75 years) and there was a roughly equal sex distribution. Nearly half of the patients died during a follow-up averaging 51.2 months (5-168 months).

Most patients demonstrated low-level proteinuria (up to 2 g/day), but no patient was at the nephrotic level. Microscopic haematuria was seen in less than one-third. Blood pressure (BP) was generally normotensive (<140/90 mmHg) or hypotensive even at the uraemic stage. Half the patients had significant renal impairment, defined as a serum creatinine level above 1.5 mg/dl and 20% eventually required renal replacement therapy. The blood urea to creatinine ratio was frequently elevated, which would be consistent with a pre-renal element to the renal impairment, which was thought to stem from volume contraction.

The majority of patients demonstrated membranoproliferative glomerulonephritis (MPGN)-like lesions as originally described by Sano *et al. *[8], characterised by marked glomerular enlargement and cellular proliferation. Some demonstrated an endarteritis of small arteries, which was unrelated to hypertension, and appeared to contribute to progressive renal damage, eventually resulting in bilateral or unilateral kidney contraction. A third of patients showed non-specific immunofluorescent staining for immunoglobulin heavy and light chains.

## Case presentation

A 46-year-old Caucasian woman was referred to our endocrinology clinic with a history of thyroxine-resistant hypothyroidism and persistent hypertension. She complained of mild numbness affecting her legs, but denied weakness. Her BP was elevated to 200/100 mmHg, and she was noted to have peripheral oedema in her shins. Blood tests revealed a mild derangement in liver function tests and a raised platelet count. A liver ultrasound confirmed no abnormalities. She was discharged from the clinic on antihypertensives and an increased dose of levothyroxine.

One year later, she was admitted with symptoms and signs of progressive peripheral motor and sensory disturbance. She had noted ascending numbness in both legs and both hands and complained of weakness in her lower legs with associated difficulty in walking.

Her examination revealed a BP of 205/108 mmHg, thickening of the skin over her hands and feet with associated peripheral oedema. There were palpable, soft rubbery lymph nodes in the cervical and submandibular region, and signs of a peripheral motor and sensory disturbance although joint position and vibration senses were well preserved. Ophthalmological examination revealed bilateral papilloedema with no haemorrhages.

Initial tests (Table 1) at presentation confirmed a mild thrombocythaemia, a mildly raised alkaline phosphatase, moderately impaired renal function (creatinine clearance 55 ml/minute) and mild-moderate proteinuria of 0.81 g/24 hours.

**Table 1:** Tests at presentation

Full blood count	Thrombocythaemia - Platelet count 520 × 10^9^/L Renal function: urea 8.2 mmol/L, creatinine 102 μmol/L, 24 hour urine collection - creatinine clearance 55 ml/minute, Protein excretion 0.81 g/24 hours
Liver function	Normal - except alkaline phosphatase 149 IU/l (normal range 38-126 IU/l) Glucose, HbA1c - normal Erythrocyte sedimentation rate (ESR) 19 mm/hour

Auto-antibodies	antinuclear factor positive - titre 1/100 (double-stranded DNA titre negative), anti-neutrophil cytoplasmic antibody (ANCA) negative, rheumatoid factor negative

Immunoglobulin levels	normal, Bence Jones Proteinuria - negative, immunofixation of serum protein electrophoresis - monoclonal band of IgA lambda

Thyroid function	free thyroxine 13.9 mIU/l (normal range 9-25 mIU/l), thyroid stimulating hormone (TSH) 6.68 mIU/l (normal range 0.5-4.5 mIU/l), thyroid peroxidase antibodies - within normal limits Growth hormones, sex hormones and cortisol levels normal 24 hour urinary catecholamines - normal

Renal ultrasound showed normal sized kidneys, and urine analysis demonstrated 3+ blood, 1+ protein, with dysmorphic red cells on urinary microscopy. Echocardiography revealed moderate left ventricular hypertrophy.

**Figure 1:**
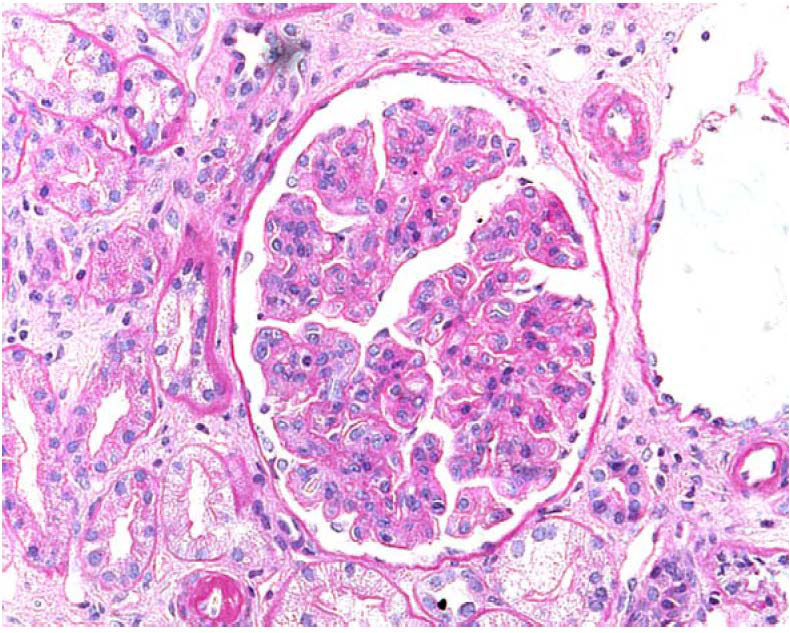
**Renal biopsy**. Periodic acid Schiff staining shows membranoproliferative/mesangiocapillary glomerulonephritis.

A renal biopsy was undertaken (Figure 1), and revealed a membranoproliferative/mesangiocapillary glomerulonephritis with endocapillary hypercellularity and obliteration or narrowing of many capillary lumens. Three out of 13 glomeruli were obsolete. Electron microscopy showed irregular reduplication of the basement membrane. There was mild hyaline thickening of small arteries and arterioles. Electron microscopy identified marked subendothelial widening with a fine granular material. IgM outlined some of the peripheral lobules and there was very variable and mild deposition of C3, but no IgG, IgA nor C1q deposition.

Computed tomography (CT) scans of the patient's chest and abdomen showed widespread but small lymphadenopathy in the thorax and axilla, and para-aortic area, with splenomegaly. Subsequent cervical lymph node biopsy showed only mild sinus histiocytosis. Trephine bone marrow biopsy revealed histologically normal marrow, with normal lymphocyte subsets on phenotyping. Biopsy of the sural nerve revealed an active neuropathy with prominent inflammatory features. There was evidence of axonal degeneration and demyelination.

A working diagnosis of POEMS syndrome was considered as the patient fulfilled most of the criteria at the time, with the exception of a paraprotein band [2]. In the light of the neuropathy, it was decided to initiate a 5-day course of intravenous immunoglobulins, and low-dose steroids (oral prednisolone 10 mg/day) were also started. Her follow-up was transferred to our hospital.

On initial presentation to us, the patient's BP was elevated (systolic BP >140 mmHg), however, following this, her BP was better controlled with systolic values in the range 110-120 mmHg and diastolic pressures in the range 60-70 mmHg.

**Figure 2:**
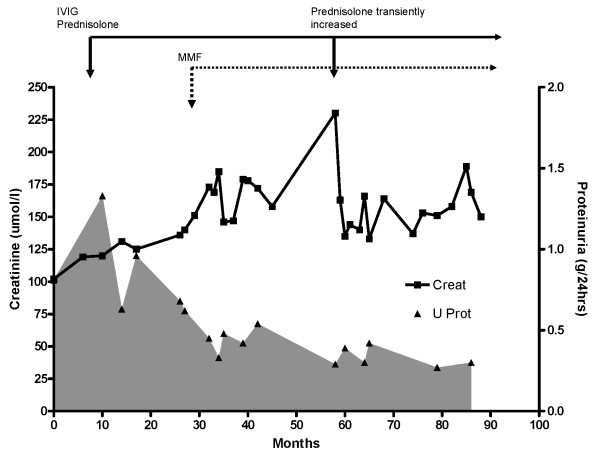
**Plasma creatinine and urinary protein concentration with time**. Plasma creatinine stabilises whilst urinary protein loss drops significantly then plateaus at ~0.3 g/24 hours.

On 10 mg of prednisolone daily, her proteinuria initially rose to 1.5 g/24 hours, but settled to approximately 0.7-0.8 g/24 hours, however, her renal function continued to deteriorate (Figure 2). Nerve conduction studies suggested a progression of her neuropathy. At this stage (just under 2 years from presentation with nephropathy), it was decided to add in low-dose mycophenolate mofetil (MMF) (250 mg twice a day) and to double the steroid dose. Since then, her renal function has remained reasonably stable, and proteinuria minimal, despite a steady reduction in prednisolone dose to a maintenance level of 7.5 mg/day.

There was a minor unexpected rise in her serum creatinine 2 years later, which promptly reversed with a transient rise in steroid dose to 20 mg/day. Around this time, she also began to develop increasingly abnormal liver function tests with rising gamma glutamyl transpeptidase (GT), alkaline phosphatase and alanine aminotransferase (ALT) - which prompted a liver biopsy. This only demonstrated nodular degenerative hyperplasia, and interestingly, the abnormal liver tests also partially resolved with the transient increase in steroid dose.

The neuropathy appears to have stabilised, and the patient remains well 6 years after initial presentation, with stable moderate renal impairment, mild proteinuria and stable moderately impaired liver tests.

Recently, using immunofixation, we have demonstrated a monoclonal band of IgA lambda; our patient finally fulfilling the criteria for POEMS syndrome.

## Discussion

A logical approach to the general treatment of POEMS is defined by the presence or absence of a dominant sclerotic plasmacytoma; in which case the first-line therapy should include radiation to the lesion. Patients with diffuse sclerotic lesions or absence of any bony lesion, and patients who have not demonstrated stabilisation of their disease by 3 to 6 months after completing radiation therapy, should be considered for systemic therapy. Treatments include corticosteroids, low-dose alkylator therapy, and high-dose chemotherapy with peripheral blood stem cell transplant [9].

Treatment options for the nephropathy of POEMS syndrome are limited and based on anecdotal case reports. Navis *et al. *[6] showed that a 20-month course of prednisolone improved the renal function of their patient although renal function did not return to normal. Erbslöh-Möller *et al. *[10] also showed that renal function improved with methylprednisolone although it rapidly deteriorated once steroid therapy was stopped.

Of the 52 patients reviewed by Nakamoto *et al. *[7], the majority received melphalan and prednisolone therapy as the baseline regimen, and occasionally additional cytotoxic drugs and steroid pulsed therapy.

More recently, Sanada *et al. *[11] treated a 61-year-old woman with POEMS syndrome characterised by advanced renal impairment with high-dose melphalan therapy (HDT) supported by autologous blood stem cell transplantation (SCT), effecting a clinical remission and striking histological resolution of renal lesions (membranoproliferative glomerulonephritis, microangiopathic glomerulopathy, and mesangiolytic lesions) on repeat biopsy. The treatment also markedly decreased serum levels of vascular endothelial growth factor (VEGF), which has been implicated in the pathogenesis of the condition, although Nakamoto *et al. *have postulated that raised VEGF levels may be a consequence rather than a cause of nephropathy [7]

MMF is a non-competitive reversible inhibitor of inosine 5-monophosphate dehydrogenase and is licensed in the UK for the prophylaxis of acute renal, cardiac and hepatic transplant rejection. Its successful use has also been described in the treatment of other immune-mediated conditions including improvement in neurological symptoms in chronic inflammatory demyelinating polyradiculopathy, and the pulmonary hypertension of POEMS syndrome.

Our patient's BP was only high at presentation with nephropathy and since then has always been well controlled (around 110-120/60-70 mmHg on most occasions). However, despite achieving this good control, her renal function continued to decline until 2 years later, when specific medication (MMF) was introduced. If the control of the BP had been the relevant intervention, one might have expected the nephropathy to stabilise sooner (perhaps after a lag phase of a few months), and we postulate that the combination of MMF and steroids is responsible for the stabilisation.

### Patient's perspective

I originally went to the GP with repeated migraines over a short period of time. I had suffered from them for some years before, but never with this frequency. The doctor found my blood pressure to be extremely high and arranged a number of blood tests which in turn revealed abnormalities in a number of the results. An appointment was then arranged with the endocrinologist as my thyroid was one of the problems. I was subsequently referred to the neurology department as by that time I was experiencing increasing numbness in my legs. By this time, I had become quite disabled and had to cease driving as I had lost the movement in one ankle. After a stay in The Royal Free Hospital as an in-patient (the admission was delayed for several months due to bed shortages), POEMS was diagnosed. That was 7 years ago. The local occupational therapist visited me at home and immediately fast tracked an application for a Disabled Badge which has been invaluable. The initial prescription of prednisolone gave me the movement back in my ankle and a great improvement in my energy levels after just a few weeks and I was able to resume driving. Since then, I have been able to live a more or less normal life with just a few restrictions due to the numbness (neuropathy) in my legs. I cannot for example, crouch or kneel down and if I do fall, I cannot get up without a great deal of help. I can also only walk short distances and only on level, smooth ground and this is further restricted if the weather is cold. I have frequent blood tests and have had variations in my drugs according to the results and have had to make some adjustments to my diet to balance out the reduced function in my kidneys. However, I still work part time, run a house and do some light gardening and I generally feel well with perhaps just more tiredness than would be normal for my age.

## Conclusion

Our patient has kept well on relatively low doses of MMF and prednisolone, with stabilisation of her nephropathy and neuropathy. We describe an alternative therapeutic option in patients with this serious but poorly understood condition. We acknowledge however that more research is required to determine whether this treatment regimen is generally appropriate for the nephropathy of this complex disorder.

## Abbreviations

ALT: alanine aminotransferase; BP: blood pressure; BUN: blood urea nitrogen; CT: computed tomography; GP: general practitioner; GT: glutamyl transpeptidase; HDT: high-dose melphalan therapy; M-band: skin changes; MMF: mycophenolate mofetil; POEMS: polyneuropathy, organomegaly, endocrinopathy; MPGN: membranoproliferative glomerulonephritis; SCT: stem cell transplantation; VEGF: vascular endothelial growth factor.

## Competing interests

The authors declare that they have no competing interests.

## Authors' contributions

GR examined the case file, reviewed literature on POEMS and made a major contribution to writing the manuscript. PW also examined the case file, made major contributions to the manuscript and interpreted the renal function data. PGR performed a histological examination of the kidney and provided a report on the pathology. MG examined the case file, contributed to the writing of the manuscript and prepared it for publication. All authors read and approved the final manuscript.

## Consent

Written informed consent was obtained from the patient for publication of this case report and any accompanying images. A copy of the written consent is available for review by the Editor-in-Chief of this journal.

## References

[B1] Nakanishi T Sobue I Toyokura Y Nishitani H Kuroiwa Y Satoyoshi E Tsubaki T Igata A Ozaki Y The Crow-Fukase syndrome: a study of 102 cases in Japan Neurology 1984 34 6 712 720 6539431 10.1212/wnl.34.6.712

[B2] Dispenzieri A Kyle RA Lacy MQ Rajkumar SV Therneau TM Larson DR Greipp PR Witzig TE Basu R Suarez GA Fonseca R Lust JA Gertz MA POEMS syndrome: definitions and long-term outcome. javascript:AL_get(this, 'jour', 'Blood.') Blood 2003 101 7 2496 2506 10.1182/blood-2002-07-2299 12456500

[B3] Seida A Wada J Morita Y Baba M Eguchi J Nishimoto N Okino T Ichimura K Yoshino T Makino H Multicentric Castleman's disease associated with glomerular microangiopathy and MPGN-like lesion: does vascular endothelial cell-derived growth factor play causative or protective roles in renal injury? Am J Kidney Dis 2004 43 1 E3 9 10.1053/j.ajkd.2003.09.023 14712466

[B4] Fakutsu A Tamai H Nishikawa K Matsukawa W Yoshida F Matsuo S Takeda A Kodera K Morozumi K Ito Y Miyakawa K Toriyama T Kawahara H Shigematsu H The kidney disease of Crowe-Fakuse (POEMS) syndrome: a clinico-pathological study of four cases Clin Nephrol 1991 36 2 76 82 1934663

[B5] Driedger H Pruzanski W Plasma cell neoplasia with peripheral polyneuropathy. A study of five cases and a review of the literature Medicine 1980 59 301 310 10.1097/00005792-198007000-00005 6248719

[B6] Navis G Dullart R Vellenga E Elema J de Jong P Renal disease in POEMS syndrome: report on a case and review of the literature Nephrol Dial Transplant 1994 9 1477 1481 7816264

[B7] Nakamoto Y Imai H Yasuda T Wakui H Miura AB A spectrum of clinicopathological features of nephropathy associated with POEMS syndrome Nephrol Dial Transplant 1999 14 2370 2386 10.1093/ndt/14.10.2370 10528660

[B8] Sano M Terasaki T Koyama A Narita M Tojo S Glomerular lesions associated with the Crow-Fukase syndrome Virchows Arch [A] 1986 409 3 9 10.1007/BF00705402 3085338

[B9] Dispenzieri A Gertz MA Treatment of POEMS syndrome Curr Treat Options Oncol 2004 5 3 249 257 10.1007/s11864-004-0016-4 15115653

[B10] Erbslöh-Möller B Perras B Sack K POEMS syndrome with chronic renal failure Med Klin 1999 94 3 159 164 10.1007/BF03044846 10218350

[B11] Sanada S Ookawara S Karube H Shindo T Goto T Nakamichi T Saito M Matsubara M Suzuki M Marked recovery of severe renal lesions in POEMS syndrome with high-dose melphalan therapy supported by autologous blood stem cell transplantation Am J Kidney Dis 2006 47 4 672 679 10.1053/j.ajkd.2006.01.004 16564945

